# Acoustic Source Localization in CFRP Composite Plate Based on Wave Velocity-Direction Function Fitting

**DOI:** 10.3390/s23063052

**Published:** 2023-03-12

**Authors:** Yu Zhang, Yu Feng, Xiaobo Rui, Lixin Xu, Lei Qi, Zi Yang, Cong Hu, Peng Liu, Haijiang Zhang

**Affiliations:** 1 State Key Laboratory of Precision Measurement Technology and Instrument, Tianjin University, Tianjin 300072, China; 2 Beijing Institute of Spacecraft Environment Engineering, Beijing 100094, China; 3 Department of Computer Science, Brown University, Providence, RI 02912, USA; 4 Guangxi Key Laboratory of Automatic Detecting Technology and Instruments, Guilin University of Electronic Technology, Guilin 541004, China; 5 Tianjin Institute of Aerospace Mechanical and Electrical Equipment, Tianjin 300450, China; 6 China Academy of Space Technology, Beijing 100094, China

**Keywords:** composite material, velocity-direction function, time difference matrix, localization

## Abstract

Composite materials are widely used, but they are often subjected to impacts from foreign objects, causing structural damage. To ensure the safety of use, it is necessary to locate the impact point. This paper investigates impact sensing and localization technology for composite plates and proposes a method of acoustic source localization for CFRP composite plates based on wave velocity-direction function fitting. This method divides the grid of composite plates, constructs the theoretical time difference matrix of the grid points, and compares it with the actual time difference to form an error matching matrix to localize the impact source. In this paper, finite element simulation combined with a lead-break experiment is used to explore the wave velocity-angle function relationship of Lamb waves in composite materials. The simulation experiment is used to verify the feasibility of the localization method, and the lead-break experimental system is built to locate the actual impact source. The results show that the acoustic emission time-difference approximation method can effectively solve the problem of impact source localization in composite structures, and the average localization error is 1.44 cm and the maximum localization error is 3.35 cm in 49 experimental points with good stability and accuracy.

## 1. Introduction

Composite materials are common and widely used in the automotive and aerospace industries [1,2,3] because of their advantages, such as high specific strength, high specific stiffness, and being lightweight.

However, after the impact of external objects on the composite structure, damage such as fracture and delamination may occur in the composite structure. Although delamination is a critical process in the operation of composite materials [4], once such damage occurs in the composite structure, the properties of the damage location will fail, and the failure site will pose a threat to the structural safety of the overall structure [5,6]. Therefore, it is necessary to locate the impact location of composite materials in time to maintain the integrity of their structural properties.

At present, the main detection methods around the impact source localization problem focus on the infrared imaging method [7,8], resistive thin film method [9], fiber grating method [10,11], acceleration method [12], and the acoustic detection method [13,14,15]. Among them, acoustic detection methods have been widely focused on due to the advantages of high system integration, high sensitivity, and fast detection speed.

In the field of acoustic detection methods, the Time Difference Of Arrival (TDOA) method is mainly used for the localization of sudden-type impact signals. The conventional TDOA method uses the time difference between the arrival of acoustic signals emitted from the target source to different sensors for localization. However, the application scenarios that need to be localized are often complex in structure, and for the problem of low localization accuracy on complex structures, various researchers have proposed improved TDOA methods.

Wang et al. [16] proposed an Adaptive Energy Compensated Threshold Filtering (AECTF) method based on acoustic emission, which can achieve large-scale, fast, and accurate localization of impact sources in reinforced slabs with less resource consumption. Qi et al. [17] proposed an algorithm for impact localization of reinforced structures based on posterior probability correlation, which combines the Gaussian correlation likelihood weighting method and the Bayesian posterior probability method to further optimize the localization results. Baxter et al. [18] proposed a delta-T mapping method for the problem of difficult localization of acoustic sources on complex structures by dividing a grid in the relevant region and performing acoustic emission experiments at the grid points to create a training library for new acoustic signal localization. This method does not need to know the wave velocity of the acoustic signal in the material, but the sensor changes its position and requires a new pre-experiment to create a new training library.

The above impact source localization methods are mainly for metallic materials; unlike metallic materials which are isotropic, carbon fiber materials are anisotropic. Carbon fiber composites, whose fibers are often arranged along the in-plane direction, can give full play to the characteristics of high fiber mechanical strength; their structural anisotropy leads to anisotropy in signal transmission. Therefore, there are effects such as attenuation and reflection that increase the difficulty of analyzing and processing signals, further reducing monitoring efficiency and accuracy. They bring new challenges for impact source localization. Various solutions have been proposed for the detection difficulties caused by anisotropy.

Kundu et al. [19,20,21,22] propose various solutions to the localization problem of anisotropic materials. They arranged multiple L-shaped sensors in composite plates, based on elliptical and parametric curve wavefront shapes, to achieve impact source localization without assuming that the signal propagates in the composite plate in a straight line and without knowing the plate properties. However, this localization system requires a large number of sensors, and the system is complex. In addition, because the symmetry axis is an unknown parameter for solving the impact source, the localization accuracy is low.

Rahim et al. [23] proposed a probability-based impact localization method for plate structures. An error index based on the arrival time of impact-induced bending waves is introduced, and an error index is introduced for each pair of sensors that lie approximately on a straight line at the grid point to determine the probability of impact localization at the grid point. For anisotropic materials, accurate knowledge of the wave velocity in all propagation directions is not required. However, this method requires that the impact point and at least two sensors lie approximately on a straight line, thus requiring the arrangement of multiple sensors and resulting in a difficult impact detection system.

Yuan et al. [24,25,26] applied the Multiple Signal Classification (MUSIC) algorithm to the impact localization of composite materials and studied an impact localization algorithm based on near-field multi-signal classification by improving the calculation of the delay matrix in the MUSIC algorithm to compensate for the wave velocity deviation caused by anisotropic materials to achieve the impact localization of composite materials. However, this localization algorithm is mainly used for near-field localization, and the impact location is assumed to be not far away from the sensor, an assumption that is difficult to meet in reality.

The above-mentioned studies have problems such as complex localization systems, low localization accuracy, or the realistic situation of the prerequisite assumptions that are difficult to satisfy for the localization of anisotropic composite plate structures. Therefore, this paper proposes an acoustic source localization method based on wave velocity-direction function fitting for a Carbon Fiber-reinforced Polymer (CFRP) composite plate. It divides the composite plate into a grid, constructs a theoretical time difference matrix of the grid points by establishing the wave velocity-direction function relationship of the acoustic signal in the plate, and compares it with the actual time difference to form an error matching matrix to localize the impact source. The location method designed in this paper solves the problem of impact source location in composite plate structures with a simple system, a small amount of data, and high stability.

## 2. Acoustic Source Localization Method

**Figure 1:**
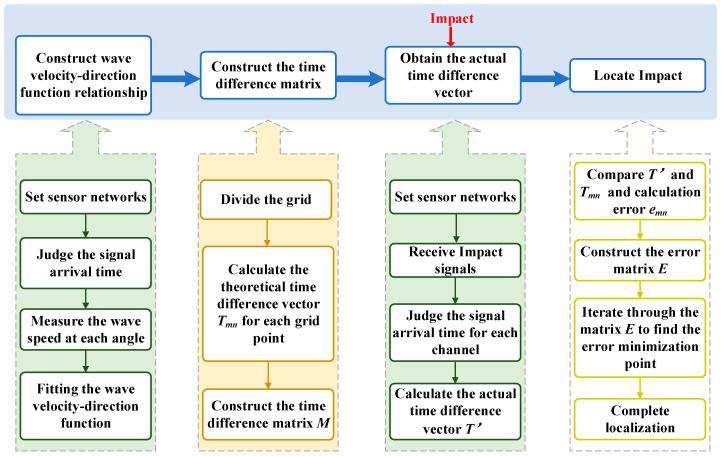
Impact localization flow chart.

As shown in Figure 1, the acoustic source localization in a CFRP composite plate based on a wave velocity-direction function fitting proposed in this paper can be divided into four processes: wave speed-direction function relationship establishment; time difference matrix establishment; the actual time difference vector obtained; and impact source localization.

**Step 1:** Construct the wave velocity-direction function relationship.

When the acoustic signal propagates in the plate structure, it usually propagates in the form of Lamb waves. Due to the anisotropy of the composite material, the acoustic signal wave velocity changes with the alteration of direction when it propagates in the composite structure, i.e., there is a certain mathematical relationship between the two. Therefore, the wave velocity-direction *V* function of the specified composite plate structure can be expressed in general terms as follows:

(1) $$V=v_{0}(\theta ).$$

As the Lamb wave propagates in the plate structure, the dispersion phenomenon occurs, generating multiple modes, and the wave speed of each mode varies in different frequency bands. Therefore, the average wave speed-direction function in a certain frequency band can be expressed as follows:

(2) $$V=\frac{\int_{fa}^{fb}v_{0}\left(\;\theta ,\;M,f\right)df}{f_{b}-f_{a}},$$

where *f* represents the frequency, *f_a_* is the lower limit of the selected frequency band, *f_b_* is the lower limit of the selected frequency band, and *M* represents the selected signal mode. Since the wave speed of each mode will change with the increase in frequency, we need to choose a narrower frequency band to filter the signal and ensure that the wave speed of the mode in the band is more stable.

Once a stable frequency band and mode are selected, the wave velocity of the signal can be measured. Construct the wave velocity-direction function *V* of the actual acoustic signal by measuring the wave velocity in each direction and then fitting it to the process:

(3) V=v(θ).

To obtain the wave velocity in each direction, the sensor network is arranged as follows: The network takes the center of the composite plate as the origin, and divides the angle evenly from 0° to 360°, and arranges the two sensors in each direction in turn. The sensor network acquires the acoustic signals, the filter performs narrow-band filtering, and finds the arrival moment of the signal by the adaptive thresholding method. Based on the relationship between time difference and distance, the velocity *V*(*θ*) is obtained and the velocity direction function relationship is fitted.

**Step 2:** Establish the simulated arrival time difference matrix.

After obtaining the wave velocity-direction function relationship, information on the time of propagation of the impact signal appearing at any point on the composite plate to the sensor position can be obtained. Thus, the composite plate can be divided into uniform grid points.

Assume that each grid point is an impact source and that the grid point coordinates and the sensor coordinates are known. The distance *L_xy_* from the grid point to each sensor can be expressed as:

(4) $$L_{xy}=\sqrt{{\left(l_{x}-x_{n}\right)}^{2}+{\left(l_{y}-y_{n}\right)}^{2}},$$

where *l_x_* is the transverse coordinate of the sensor; *l_y_* is the longitudinal coordinate of the sensor; *x_n_* is the transverse coordinate of the grid point; and *y_n_* is the longitudinal coordinate of the grid point. Therefore, the angle can be obtained by the following equation:

(5) $$\theta =\arctan\left(\frac{l_{y}-y_{n}}{l_{x}-x_{n}}\right).$$

According to Equations (3)–(5), the theoretical time *t_i_* from any grid point to the *i*-th sensor can be expressed as follows:

(6) $$t_{i}=\frac{L}{v\left(\theta \right)}.$$

The theoretical time difference $\Delta t_{ij}$ between any two sensors, *i*-th and *j*-th, can be found based on the theoretical time from the stated grid point to the sensor.

(7) $$\Delta t_{ij}=t_{i}-t_{j}\;(i\neq j),$$

then the time difference of the impact signal propagating from a point to sensor *i*-th and sensor *j*-th can be expressed as follows:

(8) $$\Delta t_{ij}=\frac{L_{i}}{v\left(\theta_{i}\right)}-\frac{L_{j}}{v\left(\theta_{j}\right)}\;(i\neq j).$$

Theoretically, the conventional time difference localization method needs to obtain at least two sets of time differences to achieve the localization of the impact source, but more sensors are usually arranged to obtain more time differences to ensure the stability of localization. Assuming that *x* sensors are arranged on the experimental structure, *x* × (*x* − 1) sets of time differences can be measured. The time difference of each grid point arriving at any two sensors is constructed as a time difference vector *T_mn_*:

(9) $$T_{mn}=\left[\begin{array}{cccc} \Delta t_{12} & \Delta t_{13} & \cdots & \Delta t_{ij} \end{array}\right]\;(i\neq j),$$

*T_mn_* denotes the full-time difference between the grid points at the *m*-th row and *n*-th column position to reach any two sensors, and the time difference vectors of all grid points are constructed into a characteristic index matrix. Thus, the index matrix of *M* is built as follows:

(10) $$M=\left(\begin{array}{ccc} T_{11} & \ldots & T_{1n}\\ \vdots & \ddots & \vdots \\ T_{m1} & \cdots & T_{mn} \end{array}\right).$$

**Step 3:** Obtain the actual time difference vector.

Four sensors are symmetrically arranged on the composite plate to form a sensor network, and when an impact signal is generated at an unknown location, the sensors receive the signal. Then the acquisition system filters the signals with a narrow band. The arrival time of the filtered signal is determined by an adaptive threshold method and the actual arrival time difference vector *T*′ is calculated as follows:

(11) $$T^{\prime }=\left[\begin{array}{cccc} {\Delta t}_{12}^{\prime } & {\Delta t}_{13}^{\prime } & \cdots & {\Delta t}_{ij}^{\prime } \end{array}\right]\;(i\neq j).$$

**Step 4:** Locate the source of the impact.

The vector *T*′ and the index matrix *M* are matched for minimum error, and the error value *e_mn_* of the time difference vector *T*′ and any vector *T_mn_* in the index matrix can be expressed as follows:

(12) $$e_{mn}=\sum_{i=1}^{x-1}\sum_{j=i+1}^{x}\left|{\Delta t}_{ij}^{\prime }-\Delta t_{ij}\right|.$$

After traversing all matrix elements, the error calculation of all grid point positions was also completed. Based on the calculated matching error results, the matching error matrix *E* can be expressed as follows:

(13) $$E=\left(\begin{array}{ccc} e_{11} & \ldots & e_{1n}\\ \vdots & \ddots & \vdots \\ e_{m1} & \cdots & e_{mn} \end{array}\right).$$

According to the result of error matching, the coordinate position of the element with the smallest matching error can be considered the location of the impact source.

## 3. Wave Speed-Direction Function Relationship Establishment

To investigate the velocity-directional function relationship of Lamb waves in composite plate structures, this experiment first analyzed the use of finite element simulation combined with an experimental approach.

### 3.1. Model Construction

#### 3.1.1. Finite Element Simulation

**Figure 2:**
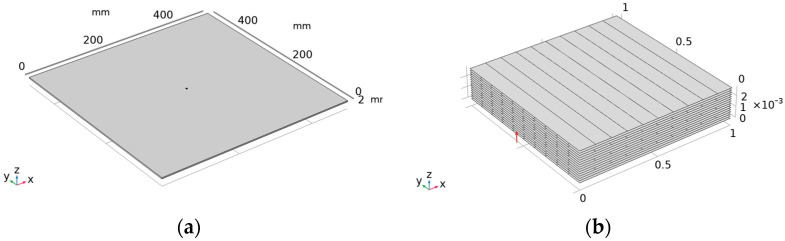
Finite element simulation model: (**a**) Cross−sectional layer preview; (**b**) Layer stacking preview. The red arrow points to the z−axis direction.

The geometric configuration is created as a rectangular body with geometric parameters of 500 mm × 500 mm × 3 mm (all simulation models are in mm). For the material, we chose to build a multilayer material and set the number of layers to 15 (each single layer is 0.2 mm), with the lay-up order being [0_3_/90/0_3_/90/0_3_/90/0_3_]. The composite structure as a whole is orthogonally anisotropic. According to the above geometric dimensions, the finite element simulation model is established as shown in Figure 2.

The physical parameters of the composite plate panel specimen are used as the basis for creating the material in the material module, and the physical parameters of the carbon fiber material used are shown in Table 1. The material properties are assigned to the rectangular structure.

The signal is emitted at the center of the upper surface of the composite plate (250, 250, and 3). The signal used in this simulation is a sinusoidal signal with a center frequency of 100 kHz, 5 periods, and a Hanning window.

**Figure 3:**
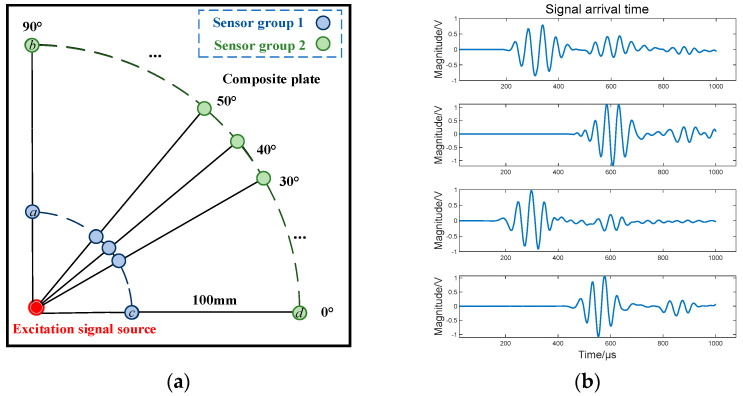
Sensor arrangement in the first quadrant and signal images of the corresponding signal acquisition points at 0° and 90°. (**a**) Sensor cluster arrangement scheme for the first quadrant; (**b**) Signals received by each sensor: the images from top to bottom are the signal images collected at points a−d, respectively.

The signal functions are as follows:

(14) f(t)=10×sin(f×2×pi×t)×(1−cos(2×pi×f×t/6)),

with (250, 250) as the origin, concentric circles are drawn with a radius of 50 mm and a radius of 150 mm. In the range of 0° to 360°, two reception points are set in 10° steps, and the distance between the two reception points is 100 mm. Therefore, the wave speed can be calculated by obtaining the time difference of the signal arriving at the two reception points in the time domain waveform. Since the layup of the composite plate is symmetrical, the distribution of wave velocity is consistent in all quadrants, and only the first quadrant wave velocity is measured in this paper. This arrangement is shown in Figure 3.

#### 3.1.2. Experimental Platform Construction and Preliminary Signal Processing

**Figure 4:**
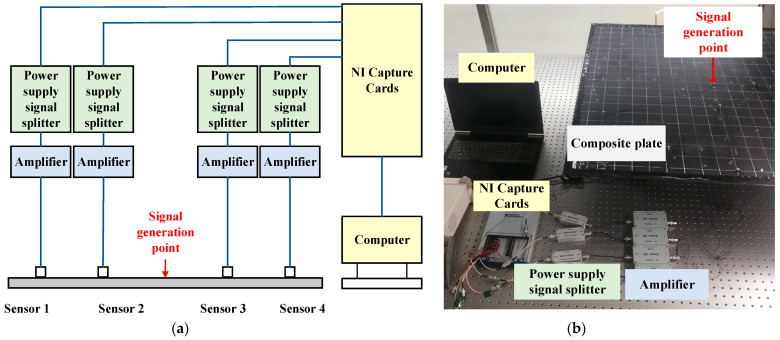
Experimental system setup: (**a**) System schematic; (**b**) Physical diagram of the system.

The impact acoustic source localization system based on wave velocity direction function fitting of a CFRP composite plate is shown in Figure 4, which consists of a carbon fiber composite plate structure model part, an acoustic emission sensor, a signal generator, a preamplifier, a signal acquisition card, and a computer. The geometry of the carbon fiber composite plate is 1000 mm × 1000 mm × 3 mm, and the material is T300 carbon fiber/IS1301 epoxy resin type composite. The lay-up method and other physical parameters are consistent with the simulation model. The edges of the plate are affixed with an acoustic-absorbing mastic to reduce the reflection effect at the edges of the plate to produce echoes affecting the measurement.

The piezoelectric transducer is a PAC Nano-30 that has a good response in the frequency band of 50~600 kHz. The preamplifier has an amplification gain of 40 dB, a bandwidth of 20–1500 kHz, and an output impedance of 50 Ω. The signal acquisition device is a USB-6366 data acquisition card manufactured by NI with a sampling rate of 2 MHz.

**Figure 5:**
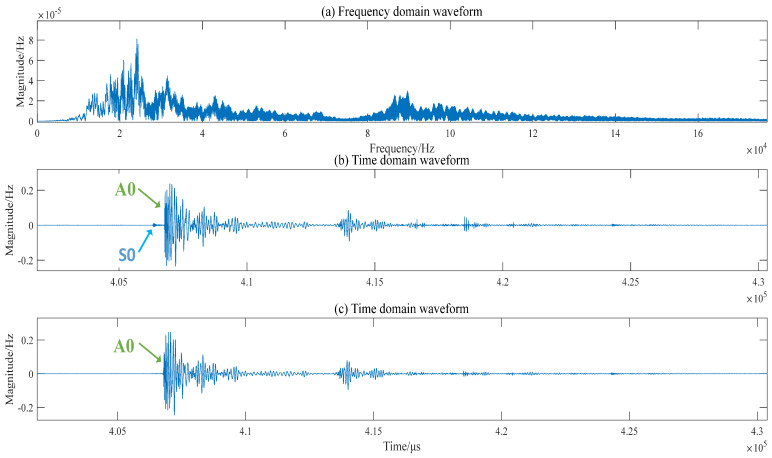
Frequency and time domain curves: (**a**) The frequency domain curve of the acoustic signal; (**b**) The time domain curves, the filtered frequency band is 20−300 kHz; (**c**)The time domain curves, the filtered frequency band is 20−100 kHz.

The obtained signal was subjected to a Fast Fourier Transform (FFT) to obtain the frequency domain curve, as shown in Figure 5a. Observing the graph, it can be seen that the energy of the signal is mainly concentrated in the low-frequency band from 0 to 100 kHz, and the energy of the signal shows oscillatory changes with the increase in frequency. Additionally, when the frequency is greater than 100 kHz, the signal energy decreases rapidly; the lead-break signal is mainly propagated in the composite plate with the low-freq signal. With the increase in propagation distance, the signal energy decreases significantly, indicating that the acoustic signal propagates into the composite plate with obvious attenuation.

Taking the time domain curve in Figure 5b as an example, the unprocessed signal waveform contains the noise signal: S0 mode and A0 mode. Since the S0 mode signal has a fast wave speed but low energy, the amplitude of the wave speed variation is large. If the arrival time difference of the S0 mode is used to calculate the wave speed, the accuracy of the location results will be reduced. Therefore, this paper mainly adopts the A0 mode signal for location. Therefore, in the subsequent filtering of the signal, we selected the filtering band of 20~100 kHz, as shown in Figure 5c, so that the S0 mode signal and the noise signal can be filtered out. The A0 mode is unaffected, and the wave velocity is not greatly affected either.

### 3.2. Wave Speed-Angle Function Relationship Establishment

According to the experimental data, the speed of the signal in a certain direction can be calculated according to the distance/time. The arrival time of the A0 signal is obtained by the adaptive threshold method, and the time difference of the signals for each sensor channel is calculated.

The arrival time difference of the signal is used to calculate the wave speed of each path, and the data for 0~90° direction are shown in Table 2:

According to the velocity fitting wave, the speed-direction curve *f(θ)* is a cubic polynomial, and *f(θ)* can be expressed as:

Simulation Model

(15) $$f(\theta )=0.0002949\theta^{3}-0.0341\theta^{2}-2.385\theta +1875,$$

Experimental model

(16) $$f(\theta )=0.0007296\theta^{3}-0.09571\theta^{2}-0.9802\theta +1745.$$

**Figure 6:**
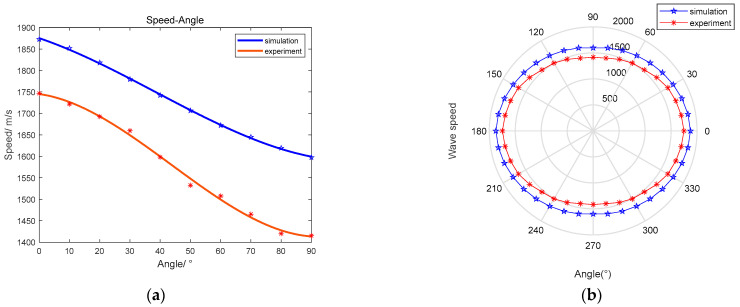
Wave speed-angle relationship. The blue curve is the simulation curve, and the red curve is the experimental curve: (**a**) Wave speed-angle cubic fitting curve; (**b**) Distribution of wave speed-angle curve in polar coordinates.

The correlation coefficient of the fitted curve for the simulation model is 0.9995, and the correlation coefficient of the fitted curve for the experimental results is 0.996. The fitted curve is shown in Figure 6a, where the blue curve is the simulation result and the red is the experimental result. The trends of the velocity curves in the simulation and the experiment are consistent, which verifies the accuracy of the simulation model. According to the results of the fitted curves, they show that the wave velocities of different paths are not constant in the composite plate structure and that the propagation velocity changes with an increase in the angle. Therefore, in determining the final location of the impact source, it is necessary to know the different locations to be combined with the location of the sensor and the wave velocity of the different propagation paths for parameter selection.

According to the orthogonal anisotropy of the composite plate and the simulation results, the wave velocity-direction curve is fitted and drawn. For a more intuitive observation of the fitting curve, the wave velocity is plotted in polar coordinates. The wave velocity-direction curve is shown in Figure 6b. The fitted curves will be used to calculate the simulated arrival time difference at the divided grid points.

## 4. Localization Results

### 4.1. Simulation Localization Results

**Figure 7:**
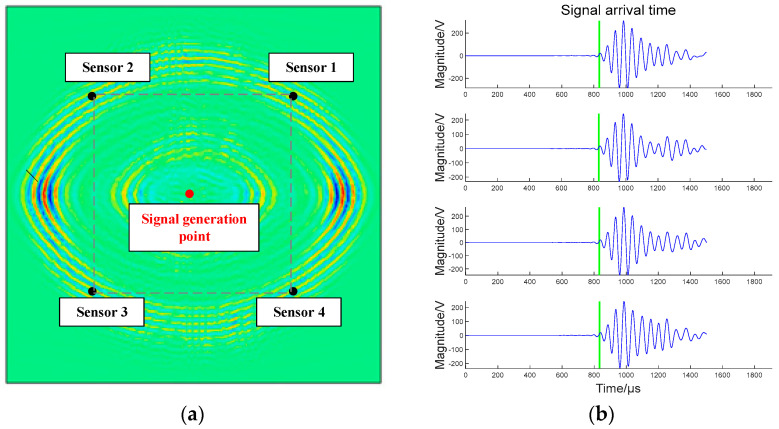
Sensor layout and signal reception: (**a**) Sensor layout and acoustic signal generation point; (**b**) Signal waveforms and arrival times of the four channels. The blue line is the acoustic signal and the green line indicates the arrival time of the signal.

Four points are evenly arranged on the four corners of the composite plate model, and the No. 1 coordinates are (300, 300), No. 2 (0, 300), No. 3 (0, 0), and No. 4 (300, 0) (unit is mm). The impact position is (150, 150). The sensors’ layout and the acoustic signal generation point are shown in Figure 7a. The signals received by the four sensors are shown in Figure 7b, and the arrival time of the signals is calibrated by the green line.

The acquisition time points of the signal arriving at the four sensors are sensors 1 (833), 2 (832), 3 (832), and 4 (833); the sample rate of the data is 5 MHz. From each of the data of the signal, the time difference between each sensor is calculated, and the data are stored in the actual time difference vector T′.

(17) $$T^{\prime }=\left[\begin{array}{cccccc} 0.0002 & 0.0002 & 0 & 0 & 0.0002 & 0.0002 \end{array}\right]\;(\mathrm{Unit}\;\mathrm{is}\;\mathrm{ms}).$$

At the same time, the computer grids the composite plate with a grid side length of 1 mm and takes each grid point as a simulated acoustic signal emitting point to calculate the theoretical time of signal arrival at each sensor according to the time difference vector of arrival and the geometric relationship. Finally, the data will be saved in matrix *M* as the index reference of the actual arrival time difference vector.

The actual time difference vector is compared with the theoretical time difference vector; the error value *e_mn_* between them is calculated, and the error matching matrix *E* is constructed. Traversing the matrix *E*, the grid point (150, 150) with the smallest matching error is the localization result of the impact source.

**Figure 8:**
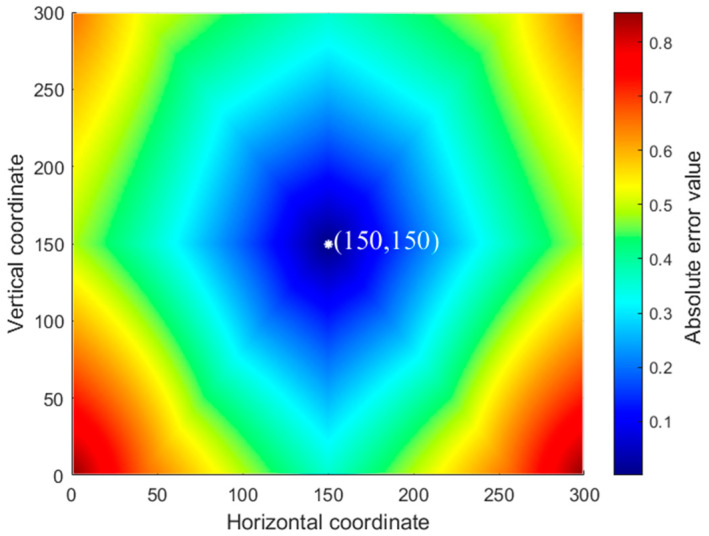
Localization result.

The error matching matrix *E* is represented graphically as shown in Figure 8, where the blue area indicates a small absolute value of the matching error and the red area indicates a large absolute value of the matching error. The graph takes the point (150, 150) as the origin, and the graph gradually transitions from blue to red during the increasing radius, which indicates that the matching error gradually increases as the grid point deviates from the impact source. Moreover, the magnitude of the error value *e_mn_* is symmetrical with the point (150, 150) as the origin, which coincides with the nature of the wave speed-angle with symmetry in the four quadrants detected in Section 2, and is also related to the symmetrical placement of the sensor in the composite plate.

The localization results verify the feasibility of the acoustic source localization method for CFRP composite plates based on the fitting of the wave velocity-direction function.

### 4.2. Lead Break Experiment Localization Results

**Figure 9:**
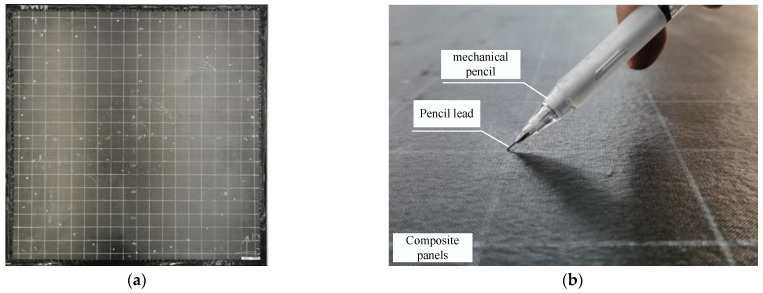
Lead breakage experiment: (**a**) Composite plate molding; (**b**) Schematic diagram of the lead breakage experiment.

When the composite plate encounters an impact, the location system receives the acoustic signal generated by the impact and processes the signal for filtering, calculation, and analysis to obtain the location of the impact source. Since the real impact may cause irreversible damage to the experimental plate, which makes it impossible to conduct repeated experiments to verify the stability of the location system, this experiment uses the broken lead signal to simulate the acoustic emission signal mode caused by the hypervelocity impact for impact source location. The lead-break experiment has the following advantages: The position of the impact can be precisely controlled. The experiment can be repeated, and the loss of the experimental specimen is small. The schematic diagram of the lead breakage experiment is shown in Figure 9.

Four acoustic emission sensors are uniformly arranged on the four corners of the composite plate at the coordinates of sensors No. 1 (800, 800), No. 2 (0, 800), No. 3 (0, 0), and No. 4 (800, 0) (the above data are in mm) to receive the acoustic signal from the impact source.

To verify the reliability of the algorithm, 49 impact points are designed for this experiment, and each point is experimented with twice; the results of the two experiments are averaged. The calculated impact source coordinates are compared with the actual impact source coordinates, and the absolute value of the error between the two points is calculated.

**Figure 10:**
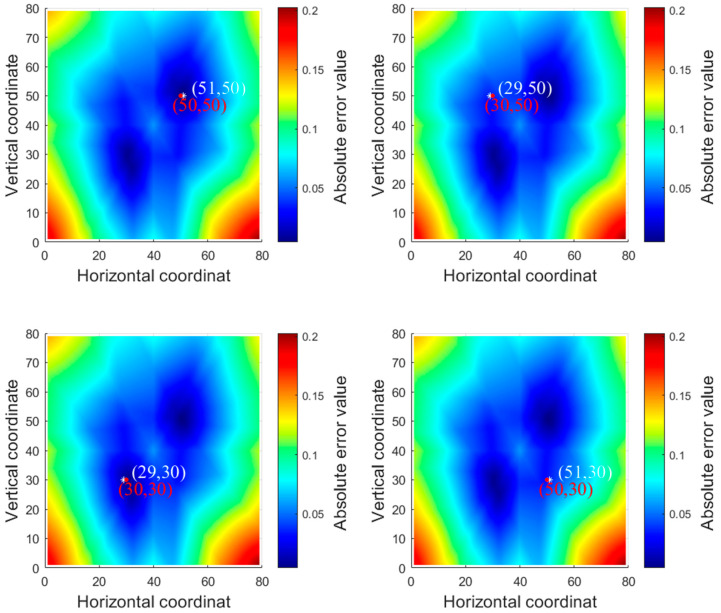
Some of the localization results. The four points are the localization results of points (50, 50), (30, 50), (30, 30), and (50, 30), with red · indicating the actual acoustic source signal and white * indicating the localization result.

Some of the point localization results are shown in Figure 10, where red · indicates the actual impact source and white * indicates the localization result.

Observing the localization of the impact source at the non-center point, two symmetrical blue peaks and valleys appear in the indexing process of all four points. The peaks and valleys are symmetrical with the origin as the center, and the matching error gradually increases with the two peaks and valleys reaching the peak at the edge position. This problem of two symmetric peaks and valleys appears because the arrangement of the sensor network and the wave speed-angle relationship of the acoustic signal in the composite plate are symmetric at the same time. Furthermore, the indexing comparison of the actual arrival time difference *t′_ij_* (*i* ≠ *j*) and the theoretical arrival time difference *t_ij_* (*i* ≠ *j*) is calculated by superimposing six sets of data, resulting in the indexing process appearing in symmetric two grid points with similar difference values: two peaks and valleys appear.

Some of the experimental data are shown in Table 3.

The minimum absolute error value is 0 cm, the maximum is 3.35 cm, and the average absolute error value of 49 points is 1.44 cm. The results show that the localization system is able to solve the problem of impact source localization in composite plate-type structures.

**Figure 11:**
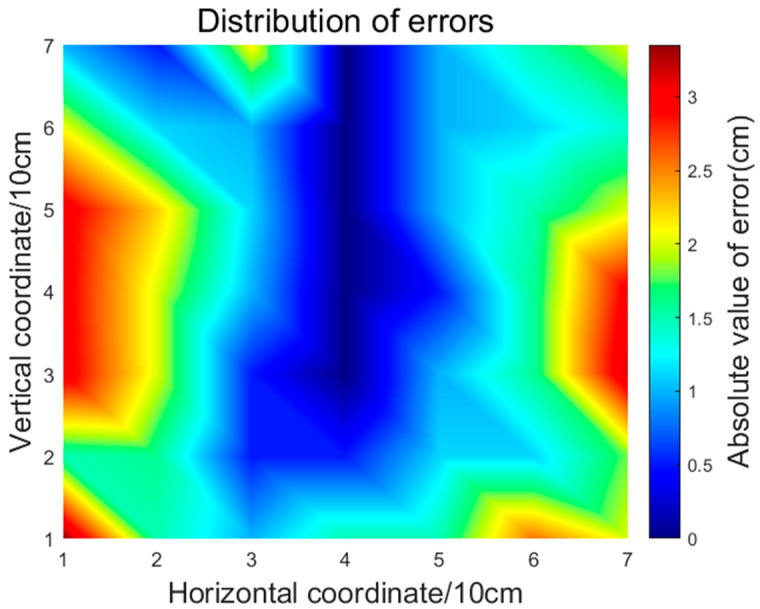
Display of the location error of all points.

To study the error distribution of the localization results, the localization results of all points are assigned to the coordinate positions of each point. The results of the error distribution are shown in Figure 11, where blue indicates a small absolute error and red indicates a large absolute error.

We compare our localization method designed in this paper with the aforementioned localization methods; the specific data are shown in Table 4. The composite plate location method based on the wave velocity-angle function fitting has the advantage of being a simple system with high accuracy.

## 5. Conclusions

Composite materials have excellent characteristics and are widely used in various industries, but their anisotropy brings challenges to impact source localization. The impact sensing and localization technology for composite plates is investigated in this study; our accomplishments in this paper are as follows:

(1) To investigate the wave velocity-angle characteristics of Lamb waves in composite plates, a finite element simulation combined with an experimental method is used. Due to the anisotropy of the composite material, the wave velocity of the acoustic signal through different paths is not constant; with the wave source as the origin and the positive direction of the x-axis to the right, the wave velocity changes as the direction changes. Overall, the shape is elliptical; thus, the three-time fitted curve of wave velocity-angle is drawn. The lead break test system is built to further verify the realism of the simulation model.

(2) This paper proposes a time difference indexing localization method to solve the problem that traditional TDOA methods cannot be applied to composite materials. The composite plate is uniformly meshed, and each grid point is assumed to be the signal source. The theoretical time difference matrix of the signal arriving at each sensor with the point as the source is calculated in turn and compared with the actual time difference to approximate the localization of the impact source. In the lead-break test, the average absolute error of the localization is 1.44 cm. The results prove that the localization system can effectively solve the impact source localization problem in the composite plate structure with good stability.

## Figures and Tables

**Table 1:** Basic physical parameters of carbon fiber composite panels.

Properties	Numerical Value	Unit
Density	1560	Kg/m³
Young’s modulus	{132 × 10^9^, 8.8 × 10^9^, 8.8 × 10^9^}	Pa
Poisson’s ratio	{0.3115,0.33,0.315}	1
Shear modulus	{4.95 × 10^9^, 3.35 × 10^9^, 4.95 × 10^9^}	N/m^2^

**Table 2:** Wave speed-angle of 0~90°.

Angle	0°	10°	20°	30°	40°	50°	60°	70°	80°	90°
Simulation speed (m/s)	1873	1852	1818	1779	1742	1706	1672	1644	1619	1597
Experimental speed (m/s)	1747	1722	1693	1660	1598	1533	1508	1465	1420	1415

**Table 3:** Some experimental data.

Experimental Coordinates/cm	Calculate Coordinates/cm	Error/cm	Experimental Coordinates/cm	Calculate Coordinates/cm	Error/cm
(10, 70)	(7, 71.5)	3.35	(30, 50)	(29.5, 50)	0.5
(20, 70)	(20, 71.5)	1.5	(40, 50)	(40, 50)	0
(30, 70)	(30, 71)	1	(50, 50)	(51, 50)	1
(40, 70)	(40, 71.5)	1.5	(60, 50)	(61.5, 50.5)	1.58
(50, 70)	(50, 71.5)	1.5	(70, 50)	(73, 51)	3.16
(60, 70)	(60.5, 72.5)	2.55	(10, 40)	(7, 40)	3
(70, 70)	(70, 72)	2	(20, 40)	(18, 40)	2
(10, 60)	(8.5, 60)	1.5	(30, 40)	(29, 40)	1
Average error	1.44 cm				

**Table 4:** Comparison of several location methods.

Method	Plate Size	Number of Sensors	Normalization Error ^1^
Elliptic and parametric curve-based positioning techniques [20]	500 × 500 mm	18	7.940 × 10^−5^ mm^−1^/10.044 × 10 ^−5^ mm^−1^
ASL technique [21]	310 × 170 mm	6	8.956 × 10 ^−5^ mm^−1^
Two-dimensional multiple signal classification method [24]	600 × 600 mm	7	2.472 × 10 ^−5^ mm^−1^
Velocity-direction function (this paper)	1000 × 1000 mm	4	1.440 × 10 ^−5^ mm^−1^

^1^ Normalization error = Average error/Plate size.

## Data Availability

The data presented in this study are not publicly available at this time, but may be obtained upon reasonable request from the authors.
